# Test-retest repeatability of child’s respiratory symptoms and perceived indoor air quality – comparing self- and parent-administered questionnaires

**DOI:** 10.1186/s12890-018-0584-x

**Published:** 2018-02-09

**Authors:** Jussi Lampi, Sari Ung-Lanki, Päivi Santalahti, Juha Pekkanen

**Affiliations:** 1 Department of Health Security, Environmental Health, National Institute for Health and Welfare, P.O. Box 95, FI-70701 Kuopio, Finland; 2 Social and Health, City of Kuopio, Kuopio, Finland; 3 Department of Health, Mental Health Unit, National Institute for Health and Welfare, Helsinki, Finland; 4 Department of Public Health, University of Helsinki, Helsinki, Finland

**Keywords:** Repeatability, Symptoms, Indoor air questionnaire

## Abstract

**Background:**

Questionnaires can be used to assess perceived indoor air quality and symptoms in schools. Questionnaires for primary school aged children have traditionally been parent-administered, but self-administered questionnaires would be easier to administer and may yield as good, if not better, information. Our aim was to compare the repeatability of self- and parent-administered indoor air questionnaires designed for primary school aged pupils.

**Methods:**

Indoor air questionnaire with questions on child’s symptoms and perceived indoor air quality in schools was sent to parents of pupils aged 7–12 years in two schools and again after two weeks. Slightly modified version of the questionnaire was administered to pupils aged 9–12 years in another two schools and repeated after a week. 351 (52%) parents and 319 pupils (86%) answered both the first and the second questionnaire. Test-retest repeatability was assessed with intra-class correlation (ICC) and Cohen’s kappa coefficients (k).

**Results:**

Test-retest repeatability was generally between 0.4–0.7 (ICC; k) in both self- and parent-administered questionnaire. In majority of the questions on symptoms and perceived indoor air quality test-retest repeatability was at the same level or slightly better in self-administered compared to parent-administered questionnaire. Agreement of self- and parent administered questionnaires was generally < 0.4 (ICC; k) in reported symptoms and 0.4–0.6 (ICC; k) in perceived indoor air quality.

**Conclusions:**

Children aged 9–12 years can give as, or even more, repeatable information about their respiratory symptoms and perceived indoor air quality than their parents. Therefore, it may be possible to use self-administered questionnaires in future studies also with children.

**Electronic supplementary material:**

The online version of this article (10.1186/s12890-018-0584-x) contains supplementary material, which is available to authorized users.

## Background

Indoor air quality problems can be caused by inadequate ventilation, unpleasant room temperature, dampness and moisture damages [1], gaseous indoor air pollutants and particulate matter [2]. At group level, for instance at work places or schools, indoor air quality problems can be assessed with indoor air questionnaires. These questionnaires typically contain questions about perceived symptoms and indoor air quality [3, 4]. However, questionnaires alone cannot verify the source or presence of indoor air problems [3]. Questionnaires can, however, point to the cause of the problem, for instance high room temperature or problems in ventilation, and therefore can be used as an aid in the problem management accompanied by adequate building and structural inspections. Indoor air questionnaires used today are validated with adult workers, but not with children [4] and are intended to be parent-administered when for example used among pupils in schools.

Questionnaires concerning child respiratory symptoms have traditionally relied on parent-report. These questionnaires have been shown to have fairly good repeatability e.g. [5–7]. However, in children’s health related quality of life questionnaires (HRQL) self-report has been increasingly used [8]. Furthermore, it has been suggested that children aged 6–11 years have basic understanding about concepts of health and have also capacity to associate health to environmental factors [9]. Questionnaires are easier to administer to pupils in schools than to their parents, which often leads to better response rates.

To our knowledge, repeatability of perceived symptoms and indoor air quality collected with self-administered questionnaires from primary school aged children has not been studied earlier. We therefore assessed the test- retest repeatability of two types of questionnaires, self- and parent-administered, on reported symptoms and perceived indoor air quality among primary school children and their parents. If it would be possible to collect reliable and repeatable information about perceived symptoms and environmental quality from the children themselves, self-administered questionnaires could be more widely used in epidemiologic studies also in younger children.

## Methods

### Study population and data collection

Present study is part of a larger study on indoor air, which was conducted in ten primary schools (age 7–12 years) in five municipalities in Finland between March 17 and April 4, 2014. All 71 regional environmental health units in Finland were approached via e-mail, asking for willingness to participate in the study and for suggestions on schools with current indoor air problems and control schools without indoor air problems. Of twelve suggestions received, five case-control school pairs were included in this study. More detailed criteria for selection of the schools are descripted previously [10]. A parent-administered questionnaire on indoor air quality was sent to all parents of the pupils in the ten schools. The research plan was approved by the Institutional Review Board (IRB) of National Institute for Health and Welfare (THL) (IRB 00007085, FWA 00014588). The study participation was voluntary and parents of the pupils were able to deny participation in advance.

Test-retest repeatability of parent-administered questionnaire was studied with a two week interval in two schools (case and control) and 351 parents (52%) returned both questionnaires. Test-retest repeatability of self-administered questionnaire was studied only among 3rd to 6th graders (age 9–12 years) with a one week interval in another two schools (case and control) and 319 pupils (86%) answered both the first and the second questionnaire. In the schools, which repeatability of self-report was tested, we had data at the baseline also from a parent -administered questionnaire, which made it possible to study also agreement between the self- and parent-administered questionnaires. However, these questionnaires were not administered exactly at the same time, as the interval between questionnaires was about 2 weeks.

### Questionnaire

Parent-administered indoor air questionnaire for primary school pupils (7–12 years) was developed to study the prevalence of self-reported symptoms and perceived indoor air quality at school. The questionnaire included two sections on symptoms in the last four weeks, categorized as respiratory symptoms (8 items) and other symptoms (17 items). All items had four response options (no; yes, sometimes; yes, every week; yes, almost every day). Every item was followed by the question “if yes, does the symptom get worse at school” with response options “no”, “yes” and “don’t know”. Symptoms were selected based on previous indoor air questionnaires used in Finland (National institute for Health and Welfare and Finnish Institute of Occupational Health) and Sweden (MM-40). The questionnaire included also questions about perceived indoor air quality at school in the last four weeks (14 items). All items had five response options (never; yes, sometimes; yes, every week; yes, almost every day; don’t know). Prevalence of asthma in the study population was determined with the question “Do your child have had following diseases in the past 12 months? Asthma?” with response options “no” and “yes”. The parents were instructed to fill in the questions on symptoms together with the child, and 89% of them reported doing so for some or most of the symptoms.

**Fig. 1:**
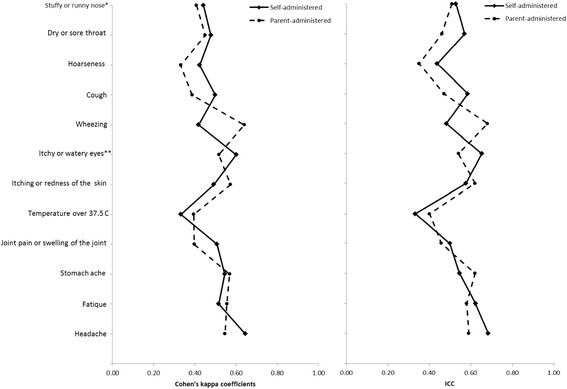
Test-retest repeatability of reported symptoms from self- and parent-administered questionnaires in primary school age children assessed with Cohen’s kappa coefficients and intra-class correlation (ICC). *Asked with two questions in parent administered questionnaire: stuffy nose (0.51 ICC; 0.41 k) and runny nose (0.38 ICC; 0.36 k). ** Asked with two questions in parent administered questionnaire: itchy eyes (0.54 ICC; 0.52 k) and watery eyes (0.41 ICC; 0.40 k)

**Fig. 2:**
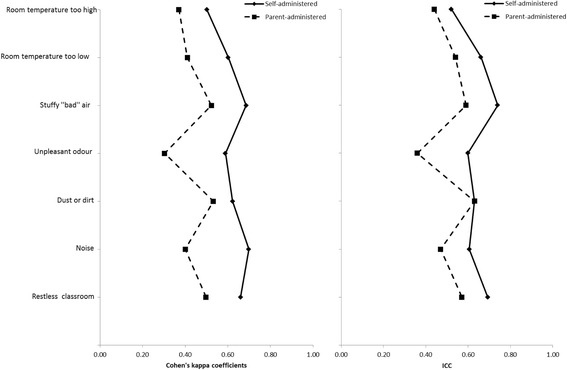
Test-retest repeatability of perceived indoor air quality and other classroom characteristics from self- and parent-administered questionnaires in primary school age children assessed with Cohen’s kappa coefficients and intra-class correlation (ICC)

Self-administered indoor air questionnaire was slightly modified version from parent-administered questionnaire. The questionnaire included respiratory symptoms (5 items) and other symptoms (7 items) in the last two weeks. All items had three response options (no; yes, sometimes; yes, almost every day) and every item was followed by the question “Does the symptom get worse at school” with response options “no/no symptom”, “yes” and “don’t know”. The questionnaire included also questions about perceived indoor air quality and other environmental characteristics at school in the last two weeks (7 items). All items had four response options (no; yes, sometimes; yes, almost every day; don’t know). Prevalence of asthma in the study population was determined with the question “Do you have asthma?” with response options “no”, “yes” and “don’t know”. Asked symptoms and environmental characteristics in schools used in both parent- and self-administered questionnaire are show in Figs. 1 and 2.

### Statistical analysis

Intra-class correlation (ICC) and Cohen’s kappa coefficients (k) and percentages of total, negative and positive agreement [11] were used to assess test-retest repeatability of self- and parent- administered questionnaire and agreement between self- and parent-administered questionnaires. As the response categories in self- and parent-administered questionnaire varied, the parent-administered answers were categorized to 3 classes (“no”, “yes, sometimes” or “yes, every week” and “yes almost every day”) for these analyses, so that the number of categories would be the same. To calculate Cohen’s kappa coefficients and percentages of total, negative and positive agreement, responses were dichotomized to “no” or “yes” (includes all of the different “yes” answer options). All of the analyses were done with SPSS.

## Results

Test-retest repeatability of parent-administered questionnaire was assessed with 351 pupils aged 7–12 years and from these pupils 49% were boys and 51% girls. Similarly, test-retest repeatability of self-administered questionnaire was assessed with 319 pupils aged 9–12 years and from these pupils 44% were boys and 56% girls. Prevalence of the symptoms, perceived indoor air quality and missing information in these two populations are show in Additional file 1: Table S1 and S2. Prevalence of missing information in symptoms and perceived indoor air quality was low, generally around 1 %, and highest percentage of missing information observed was 2.7% (wheezing in self-administered questionnaire). Highest prevalence of don’t know answers was 13.5% in self-administered questionnaire (dust and dirt) and 7.5% (unpleasant odor) in parent-administered questionnaire, but generally lower in both self- and parent- administered questionnaires. Missing information and don’t know answers were excluded from further analysis.

### Repeatability of the self-administered questionnaire

Test-retest repeatability of questions on symptoms and perceived indoor air quality in schools from self-administered questionnaires were generally within 0.4–0.7 (ICC, k) (Figs. 1 and 2). Only question with repeatability below 0.4 was temperature over 37.5 C in past 2 weeks (0.33 ICC; k). For sensitivity analysis, we stratified the analysis by pupil’s grade in schools, but no major differences in test-retest repeatability in self-administered questionnaire for symptoms and perceived indoor air quality between the younger or older pupils were observed (data not shown). Percentages of observed total agreement and proportions of negative and positive agreement of question from self-administered questionnaire are shown in Additional file 1: Table S3.

### Repeatability of the parent-administered questionnaire

Test-retest repeatability of reported symptoms and perceived indoor air quality in schools from parent-administered questionnaires were generally at the same level or slightly lower than in self-administered questionnaire and generally was within 0.4–0.7 (ICC, k) in parent-administered questionnaire also (Figs. 1 and 2). The questions with test-retest repeatability below 0.4 were runny nose (0.38 ICC; 0.36 k), hoarseness (0.35 ICC; 0.33 k), and cough (0.47 ICC; 0.39 k). Only symptom where test-retest repeatability was distinctly higher in parent-administered questionnaire was wheezing (0.68 vs. 0.48 ICC; 0.64 vs. 0.42 k). Percentages of observed total agreement and proportions of negative and positive agreement of question from parent-administered questionnaire are shown in Additional file 1: Table S4.

### Agreement between the self- and parent administered questionnaires

**Fig. 3:**
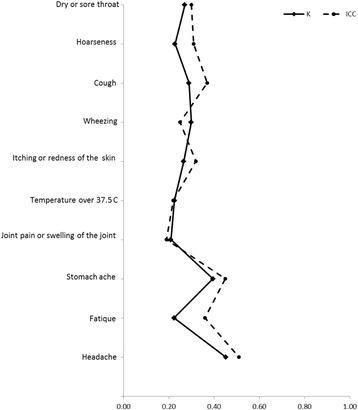
Agreement of reported symptoms between self- and parent administered questionnaires in primary school aged children assessed with Cohen’s kappa coefficient (k) and intra-class correlation (ICC)

**Fig. 4:**
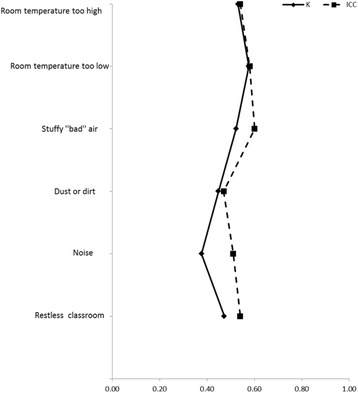
Agreement of perceived indoor air quality between self- and parent administered questionnaires in primary school aged children assessed with Cohen’s kappa coefficient (k) and intra-class correlation (ICC)

Agreement between self- and parent administered questionnaires at baseline was generally within 0.2–0.4 (ICC; k) in all reported symptoms expect headache (0.51 ICC; 0.45 k) (Fig. 3). Compared to reported symptoms, agreement between parent- and self-administered questionnaires in perceived indoor air quality was higher, as the intra-class correlation was between 0.47–0.60 and Cohen’s kappa coefficient between 0.38–0.58 (Fig. 4). Percentages of observed total agreement and proportions of negative and positive agreement of question between self- and parent administered questionnaires are shown in Additional file 1: Table S5.

### Asthma

Prevalence of asthma was 8.8% in both self-administered questionnaires and Cohen’s kappa coefficient between self-administered questionnaires was 0.98. In first self-administered questionnaire prevalence of don’t know answers was 7.6% and missing information was 0.6%. In second self-administered questionnaire prevalence of don’t know answers was 8.8% and missing information was 0.6%.

Prevalence of asthma was 9.3% in the first parent-administered questionnaire and 8.6% in the second parent-administered questionnaire. Cohen’s kappa coefficient between parent-administered questionnaires was 0.87 in reported asthma. In first parent-administered questionnaire prevalence of missing information was 2.3%. In second parent-administered questionnaire prevalence missing information was 3.4%. Cohen’s kappa coefficient between parent- and self-administered questionnaires (parent-child agreement) was 0.87 in reported asthma.

## Discussion

Present study shows that primary school aged children can give repeatable information about their symptoms and perceived indoor air quality at schools. In perceived indoor air quality and with majority of symptoms the repeatability was at the same level or higher in self-administered than parent-administered questionnaire. Agreement between the parent and child was generally poor for symptoms, but better for perceived indoor air quality.

Primary school aged children in this study could give as, or even more, repeatable information about their symptoms and perceived indoor air quality as their parents. To our knowledge, there are no previous studies comparing repeatability among parents and children. In a study by Olson et al. asthmatic children even down to 7 years old could give repeatable information with interview-administered questionnaires about their health [12]. Adolescents have also been shown to give repeatable information about their respiratory symptoms with self-administered questionnaires [13, 14]. Furthermore, it has been reported that children aged 11 years can give repeatable information about their physical and social environment at home and in neighborhood [15]. In our study, only in questions about temperature over 37.5 C and wheezing the test-retest repeatability was distinctly lower in self-administered than in parent-administered questionnaire. Wheezing is a difficult concept even for parents to understand [16, 17], therefore it is not surprising that repeatability of self-reported wheeze was poor in children. Thus, questions should be quite descriptive or should be visualized to be more understandably for children [9]. However, based on this and previous studies, children aged 9–12 years appear to be able to give repeatable information about their symptoms and perceived indoor air quality.

In this study parent-child agreement on symptoms was low, which is in line with previous studies [18–20]. It is possible that this is partly explained by characteristics of the child or the parent, which may lead to under- or over reporting of symptoms. It has been shown that mothers self-reported health is strongly associated with how she reports on the health of the child [21]. Parent’s lifestyle factors (e.g. smoking) may also affect their reporting on child’s symptoms [22]. Furthermore, psychosocial factors are known to affect environmental complaints and perceived symptoms [23]. However, agreement on perceived indoor air quality was better than for symptoms in the present study.

The validity of the information given by the children cannot be assessed with the present results and study design. Previous studies have suggested that in child perception of asthma symptoms are associated with objective measurements of the lung function and the correlation between symptoms and lung function may be better or at the same level than parent’s perception of the symptoms [24, 25] but dependent on age of the child [26]. In this study, child-parent agreement on asthma was excellent which is in line with previous studies [18, 20, 27]. This gives some suggestive indication also for validity of the questionnaire.

The test-retest repeatability of reported symptoms and perceived indoor air quality in both self- and parent-administered questionnaire was generally 0.4–0.7 (ICC, k) in this study. This level of repeatability is generally considered to be acceptable for this type of questionnaires. However, when test-retest repeatability is assessed by using kappa values, the dependence of kappa values on marginal prevalence’s (i.e. amount of change in agreement) should be acknowledged. The dependence results directly from definition of kappa [28].

The few limitations of the study should be acknowledged. The self-administered questionnaire was slightly different from the parent-administered questionnaire. There were fewer questions, questions had one less response category, and the time period used was different (2 weeks in self-reported vs. 4 weeks in parent-reported questionnaire). Furthermore, the time interval in which the test-retest was performed was not the same (1 week in self-reported vs. 2 weeks in parent-reported questionnaire). These factors can affect differences between test-retest repeatability of self- and parent-administered questionnaires and agreement between self- and parent- reported symptoms. It should also be noted that the repeatability of self and parent-administered questionnaires were tested in different schools, since otherwise the children would have had to take part in filling of four questionnaires in total, two self-administered and two parent-administered, as parent-administered questionnaires were instructed to be filled in together with the child. Furthermore, the agreement of the reported symptoms between self- and parent administered questionnaire can be partly affected by the fact that questionnaires were not administered exactly at the same time.

## Conclusion

Children aged 9–12 years can give as, or even more, repeatable information about their symptoms and perceived indoor air quality than their parents. Therefore, in future it may be possible to use self-administered questionnaires in epidemiologic studies on perceived indoor air quality and symptoms also among primary school age children. If indoor air questionnaires could be administered to pupils during school day, this could lead to better response rates and even more reliable results.

## Additional file

Additional file 1: Test-retest repeatability of child's respiratory symptoms and perceived indoor air quality - Additional file. (PDF 200 kb)

## Abbreviations

ICC: intra-class correlation
K: Cohen’s kappa coefficients
