# Supplementary material for: Test-retest repeatability of child’s respiratory symptoms and perceived indoor air quality – comparing self- and parent-administered questionnaires
Source: BMC Pulm Med. 2018 Feb 9;18:32. doi: 10.1186/s12890-018-0584-x (PMC5807794; doi:10.1186/s12890-018-0584-x)
Supplement: Supplementary file 1 — Test-retest repeatability of child's respiratory symptoms and perceived indoor air quality - Additional file. (PDF 200 kb) [file 12890_2018_584_MOESM1_ESM.pdf]

Test-retest repeatability of child's respiratory symptoms and perceived indoor air quality – Comparing self - and parent-administered questionnaires

Lampi J, Ung-Lanki S, Santalahti P, Pekkanen J

Additional file

Table S1. Prevalence of reported symptoms from self-administered (N=638) and parent-administered questionnaire (N=702) in primary school age children. Table shows numbers for the combined data using both the first and the second questionnaire.

| Reported symptoms | | Self-administered questionnaire | | | | Reported symptoms | | Parent-administered questionnaire | | | | |
|---|---|---|---|---|---|---|---|---|---|---|---|---|
| | | No | Yes, sometimes | Yes, almost every day | Missing | | | No | Yes, sometimes | Yes, every week | Yes, almoust every day | Missing |
| Stuffy or runny nose | n | 145 | 340 | 149 | 4 | Stuffy nose | n | 202 | 378 | 66 | 47 | 9 |
| | % | 22.7 | 53.3 | 23.4 | 0.6 | | % | 28.8 | 53.8 | 9.4 | 6.7 | 1.3 |
| | | | | | | Runny nose | n | 174 | 445 | 49 | 28 | 6 |
| | | | | | | | % | 24.8 | 63.4 | 7.0 | 4.0 | 0.9 |
| Dry or sore throat | n | 370 | 216 | 44 | 8 | Dry or sore throat | n | 321 | 343 | 25 | 5 | 8 |
| | % | 58.0 | 33.9 | 6.9 | 1.3 | | % | 45.7 | 48.9 | 3.6 | 0.7 | 1.1 |
| Hoarseness | n | 396 | 207 | 25 | 10 | Hoarseness | n | 458 | 210 | 20 | 4 | 10 |
| | % | 62.1 | 32.4 | 3.9 | 1.6 | | % | 65.2 | 29.9 | 2.8 | 0.6 | 1.4 |
| Cough | n | 272 | 297 | 64 | 5 | Cough | n | 293 | 352 | 31 | 20 | 6 |
| | % | 42.6 | 46.6 | 10.0 | 0.8 | | % | 41.7 | 50.1 | 4.4 | 2.8 | 0.9 |
| Wheezing | n | 560 | 47 | 14 | 17 | Wheezing | n | 642 | 49 | 2 | 1 | 8 |
| | % | 87.8 | 7.4 | 2.2 | 2.7 | | % | 91.5 | 7.0 | 0.3 | 0.1 | 1.1 |
| Itchy or watery eyes | n | 299 | 249 | 83 | 7 | Itchy eyes | n | 473 | 176 | 34 | 12 | 7 |
| | % | 46.9 | 39.0 | 13.0 | 1.1 | | % | 67.4 | 25.1 | 4.8 | 1.7 | 1.0 |
| | | | | | | Watery eyes | n | 551 | 115 | 16 | 9 | 11 |
| | | | | | | | % | 78.5 | 16.4 | 2.3 | 1.3 | 1.6 |
| Itching or redness of the skin | n | 452 | 128 | 46 | 12 | Itching or redness of the skin | n | 453 | 161 | 38 | 41 | 9 |
| | % | 70.8 | 20.1 | 7.2 | 1.9 | | % | 64.5 | 22.9 | 5.4 | 5.8 | 1.3 |
| Temperature over 37.5 C | n | 568 | 65 | 0 | 5 | Temperature over 37.5 C | n | 540 | 153 | 1 | 1 | 7 |
| | % | 89.0 | 10.2 | 0.0 | 0.8 | | % | 76.9 | 21.8 | 0.1 | 0.1 | 1.0 |
| Joint pain or swelling of joint | n | 536 | 83 | 9 | 10 | Joint pain or swelling of joint | n | 643 | 44 | 9 | 1 | 5 |
| | % | 84.0 | 13.0 | 1.4 | 1.6 | | % | 91.6 | 6.3 | 1.3 | 0.1 | 0.7 |
| Stomach ache | n | 389 | 216 | 25 | 8 | Stomach ache | n | 445 | 218 | 26 | 8 | 5 |
| | % | 61.0 | 33.9 | 3.9 | 1.3 | | % | 63.4 | 31.1 | 3.7 | 1.1 | 0.7 |
| Fatique | n | 179 | 315 | 137 | 7 | Fatique | n | 291 | 335 | 49 | 22 | 5 |
| | % | 28.1 | 49.4 | 21.5 | 1.1 | | % | 41.5 | 47.7 | 7.0 | 3.1 | 0.7 |
| Headache | n | 242 | 261 | 132 | 3 | Headache | n | 349 | 302 | 38 | 10 | 3 |
| | % | 37.9 | 40.9 | 20.7 | 0.5 | | % | 49.7 | 43.0 | 5.4 | 1.4 | 0.4 |

Table S2. Prevalence of perceived indoor air quality from self-administered (N=638) and parent-administered questionnaire (N=702) in primary school age children. Table shows numbers for the combined data using both the first and the second questionnaire.

| Perceived indoor air quality | | Self-administered questionnaire | | | | | Parent-administered questionnaire | | | | | |
|---|---|---|---|---|---|---|---|---|---|---|---|---|
| | | No | Yes, sometimes | Yes, almost every day | Don't know | Missing | No | Yes, sometimes | Yes, every week | Yes, almoust every day | Don't Know | Missing |
| Room temperature too high | n | 306 | 253 | 33 | 40 | 6 | 384 | 245 | 24 | 20 | 25 | 4 |
| | % | 48.0 | 39.7 | 5.2 | 6.3 | 0.9 | 54.7 | 34.9 | 3.4 | 2.8 | 3.6 | 0.6 |
| Room temperature too low | n | 334 | 197 | 63 | 40 | 4 | 453 | 192 | 18 | 12 | 21 | 6 |
| | % | 52.4 | 30.9 | 9.9 | 6.3 | 0.6 | 64.5 | 27.4 | 2.6 | 1.7 | 3.0 | 0.9 |
| Stuffy "bad" air | n | 188 | 252 | 126 | 72 | 0 | 361 | 204 | 41 | 37 | 51 | 8 |
| | % | 29.5 | 39.5 | 19.7 | 11.3 | 0.0 | 51.4 | 29.1 | 5.8 | 5.3 | 7.3 | 1.1 |
| Unpleasant odour | n | 350 | 206 | 34 | 47 | 1 | 491 | 140 | 5 | 7 | 53 | 6 |
| | % | 54.9 | 32.3 | 5.3 | 7.4 | 0.2 | 69.9 | 19.9 | 0.7 | 1.0 | 7.5 | 0.9 |
| Dust and dirt | n | 377 | 146 | 28 | 86 | 1 | 453 | 161 | 21 | 16 | 47 | 4 |
| | % | 59.1 | 22.9 | 4.4 | 13.5 | 0.2 | 64.5 | 22.9 | 3.0 | 2.3 | 6.7 | 0.6 |
| Noise | n | 221 | 280 | 103 | 32 | 2 | 180 | 343 | 87 | 74 | 14 | 4 |
| | % | 34.6 | 43.9 | 16.1 | 5.0 | 0.3 | 25.6 | 48.9 | 12.4 | 10.5 | 2.0 | 0.6 |
| Restlesness in the classroom | n | 232 | 266 | 94 | 45 | 1 | 156 | 372 | 73 | 74 | 22 | 5 |
| | % | 36.4 | 41.7 | 14.7 | 7.1 | 0.2 | 22.2 | 53.0 | 10.4 | 10.5 | 3.1 | 0.7 |

Table S3. Percentage of observed total agreement and proportions of negative and positive agreement of reported symptoms and perceived indoor air quality from self-administered questionnaires in primary school age children.

| Reported symptoms | N | n(1+2+) | % | n(1+2-) | % | n(1-2+) | % | n(1-2-) | % | Ppos | Pneg | Ptotal |
|---|---|---|---|---|---|---|---|---|---|---|---|---|
| Stuffy or runny nose | 315 | 212 | 67 | 30 | 10 | 32 | 10 | 41 | 13 | 87.2 | 56.9 | 80.3 |
| Dry or sore throat | 311 | 89 | 29 | 31 | 10 | 48 | 15 | 143 | 46 | 69.3 | 78.4 | 74.6 |
| Hoarseness | 310 | 73 | 24 | 31 | 10 | 53 | 17 | 153 | 49 | 63.5 | 78.5 | 72.9 |
| Cough | 314 | 141 | 45 | 36 | 11 | 41 | 13 | 96 | 31 | 78.6 | 71.4 | 75.5 |
| Wheezing | 302 | 14 | 5 | 14 | 5 | 17 | 6 | 257 | 85 | 47.5 | 94.3 | 89.7 |
| Itchy or watery eyes | 312 | 134 | 43 | 31 | 10 | 31 | 10 | 116 | 37 | 81.2 | 78.9 | 80.1 |
| Itching or redness of skin | 308 | 54 | 18 | 23 | 7 | 40 | 13 | 191 | 62 | 63.2 | 85.8 | 79.5 |
| Temperature over 37.5 C | 314 | 13 | 4 | 23 | 7 | 16 | 5 | 262 | 83 | 40.0 | 93.1 | 87.6 |
| Joint pain or swelling of joint | 309 | 26 | 8 | 14 | 5 | 24 | 8 | 245 | 79 | 57.8 | 92.8 | 87.7 |
| Stomach ache | 311 | 86 | 28 | 31 | 10 | 36 | 12 | 158 | 51 | 72.0 | 82.5 | 78.5 |
| Fatique | 312 | 194 | 62 | 35 | 11 | 26 | 8 | 57 | 18 | 86.4 | 65.1 | 80.4 |
| Headache | 316 | 169 | 53 | 28 | 9 | 25 | 8 | 94 | 30 | 86.4 | 78.0 | 83.2 |
| **Perceived indoor air quality** | **N** | **n(1+2+)** | **%** | **n(1+2-)** | **%** | **n(1-2+)** | **%** | **n(1-2-)** | **%** | **Ppos** | **Pneg** | **Ptotal** |
| Room temperature too high | 280 | 100 | 36 | 28 | 10 | 42 | 15 | 110 | 39 | 74.1 | 75.9 | 75.0 |
| Room temperature too low | 281 | 94 | 33 | 20 | 7 | 35 | 12 | 132 | 47 | 77.4 | 82.8 | 80.4 |
| Stuffy ''bad'' air | 258 | 155 | 60 | 10 | 4 | 26 | 10 | 67 | 26 | 89.6 | 78.8 | 86.0 |
| Unpleasant odour | 278 | 85 | 31 | 27 | 10 | 28 | 10 | 138 | 50 | 75.6 | 83.4 | 80.2 |
| Dust or dirt | 247 | 56 | 23 | 12 | 5 | 28 | 11 | 151 | 61 | 73.7 | 88.3 | 83.8 |
| Noise | 292 | 159 | 54 | 29 | 10 | 24 | 8 | 80 | 27 | 85.7 | 75.1 | 81.8 |
| Restless classroom | 281 | 147 | 52 | 34 | 12 | 12 | 4 | 88 | 31 | 86.5 | 79.3 | 83.6 |

n(1+2+): number of subjects with positive answer in both questionnaires; n(1-2-): number of subjects with negative answer in both questionnaires
n(1-2+): number of subject with negative answer in first questionnaire and positive answer in second questionnaire
n(1+2-): number of subject with positive answer in first questionnaire and negative answer in second questionnaire
Ppos: observed proportion of positive agreement; Pneg: observed proportion of negative agreement; Ptotal: observed proportion of total agreement

Table S4. Percentage of observed total agreement and proportions of negative and positive agreement of reported symptoms and perceived indoor air quality in schools from parent-administered questionnaires in primary school age children.

| Reported symptoms | N | n(1+2+) | % | n(1+2-) | % | n(1-2+) | % | n(1-2-) | % | Ppos | Pneg | Ptotal |
|---|---|---|---|---|---|---|---|---|---|---|---|---|
| Stuffy nose | 342 | 202 | 59 | 39 | 11 | 44 | 13 | 57 | 17 | 83.0 | 57.9 | 75.7 |
| Runny nose | 345 | 218 | 63 | 41 | 12 | 42 | 12 | 44 | 13 | 84.0 | 51.5 | 75.9 |
| Dry or sore throat | 343 | 137 | 40 | 54 | 16 | 40 | 12 | 112 | 33 | 74.5 | 70.4 | 72.6 |
| Hoarseness | 341 | 64 | 19 | 49 | 14 | 53 | 16 | 175 | 51 | 55.7 | 77.4 | 70.1 |
| Cough | 345 | 149 | 43 | 46 | 13 | 57 | 17 | 93 | 27 | 74.3 | 64.4 | 70.1 |
| Wheezing | 344 | 17 | 5 | 10 | 3 | 7 | 2 | 310 | 90 | 66.7 | 97.3 | 95.1 |
| Itchy eyes | 344 | 73 | 21 | 33 | 10 | 39 | 11 | 199 | 58 | 67.0 | 84.7 | 79.1 |
| Watery eyes eyes | 342 | 36 | 11 | 24 | 7 | 42 | 12 | 240 | 70 | 52.2 | 87.9 | 80.7 |
| Itching or redness of skin | 342 | 85 | 25 | 37 | 11 | 29 | 8 | 191 | 56 | 72.0 | 85.3 | 80.7 |
| Temperature over 37.5 C | 344 | 41 | 12 | 28 | 8 | 45 | 13 | 230 | 67 | 52.9 | 86.3 | 78.8 |
| Joint pain or swelling of joint | 346 | 12 | 3 | 16 | 5 | 14 | 4 | 304 | 88 | 44.4 | 95.3 | 91.3 |
| Stomach ache | 346 | 91 | 26 | 35 | 10 | 34 | 10 | 186 | 54 | 72.5 | 84.4 | 80.1 |
| Fatique | 346 | 164 | 47 | 46 | 13 | 29 | 8 | 107 | 31 | 81.4 | 74.0 | 78.3 |
| Headache | 348 | 135 | 39 | 44 | 13 | 35 | 10 | 134 | 39 | 77.4 | 77.2 | 77.3 |
| **Perceived indoor air quality** | **N** | **n(1+2+)** | **%** | **n(1+2-)** | **%** | **n(1-2+)** | **%** | **n(1-2-)** | **%** | **Ppos** | **Pneg** | **Ptotal** |
| Room temperature too high | 325 | 88 | 27 | 49 | 15 | 51 | 16 | 137 | 42 | 63.8 | 73.3 | 69.2 |
| Room temperature too low | 326 | 65 | 20 | 43 | 13 | 42 | 13 | 176 | 54 | 60.5 | 80.5 | 73.9 |
| Stuffy "bad" air | 302 | 97 | 32 | 31 | 10 | 40 | 13 | 134 | 44 | 73.2 | 79.1 | 76.5 |
| Other Unpleasant odour | 303 | 33 | 11 | 32 | 11 | 44 | 15 | 194 | 64 | 46.5 | 83.6 | 74.9 |
| Dust or dirt | 308 | 63 | 20 | 26 | 8 | 35 | 11 | 184 | 60 | 67.4 | 85.8 | 80.2 |
| Noise | 333 | 206 | 62 | 48 | 14 | 30 | 9 | 49 | 15 | 84.1 | 55.7 | 76.6 |
| Restless classroom | 325 | 219 | 67 | 36 | 11 | 23 | 7 | 47 | 14 | 88.1 | 61.4 | 81.8 |

n(1+2+): number of subjects with positive answer in both questionnaires; n(1-2-): number of subjects with negative answer in both questionnaires
n(1-2+): number of subject with negative answer in first questionnaire and positive answer in second questionnaire
n(1+2-): number of subject with positive answer in first questionnaire and negative answer in second questionnaire
Ppos: observed proportion of positive agreement; Pneg: observed proportion of negative agreement; Ptotal: observed proportion of total agreement

Table S5. Percentage of observed total agreement and proportions of negative and positive agreement of reported symptoms and perceived indoor air quality between self- and parent-administered questionnaires in primary school age children.

| | N | n(P+S+) | % | n(P+S-) | % | n(P-S+) | % | n(P-S-) | % | Ppos | Pneg | Ptotal |
|---|---|---|---|---|---|---|---|---|---|---|---|---|
| Dry or Sore throat | 279 | 88 | 32 | 89 | 32 | 19 | 7 | 83 | 30 | 62,0 | 60,6 | 61,3 |
| Hoarseness | 277 | 57 | 21 | 70 | 25 | 34 | 12 | 116 | 42 | 52,3 | 69,0 | 62,5 |
| Cough | 281 | 111 | 40 | 52 | 19 | 46 | 16 | 72 | 26 | 69,4 | 59,5 | 65,1 |
| Wheezing | 276 | 8 | 3 | 13 | 5 | 16 | 6 | 239 | 87 | 35,6 | 94,3 | 89,5 |
| Itching or redness of skin | 276 | 50 | 18 | 69 | 25 | 26 | 9 | 131 | 47 | 51,3 | 73,4 | 65,6 |
| Temperature over 37.5 C | 280 | 16 | 6 | 43 | 15 | 18 | 6 | 203 | 73 | 34,4 | 86,9 | 78,2 |
| Joint pain or swelling of joint | 277 | 10 | 4 | 24 | 9 | 22 | 8 | 221 | 80 | 30,3 | 90,6 | 83,4 |
| Abdominal pain | 280 | 67 | 24 | 43 | 15 | 37 | 13 | 133 | 48 | 62,6 | 76,9 | 71,4 |
| Fatique | 279 | 156 | 56 | 42 | 15 | 46 | 16 | 35 | 13 | 78,0 | 44,3 | 68,5 |
| Headache | 284 | 149 | 52 | 43 | 15 | 28 | 10 | 64 | 23 | 80,8 | 64,3 | 75,0 |
| | N | n(P+S+) | % | n(P+S-) | % | n(P-S+) | % | n(P-S-) | % | Ppos | Pneg | Ptotal |
| Room temperature too high | 254 | 84 | 33 | 24 | 9 | 35 | 14 | 111 | 44 | 74,0 | 79,0 | 76,8 |
| Room temperature too low | 257 | 79 | 31 | 35 | 14 | 18 | 7 | 125 | 49 | 74,9 | 82,5 | 79,4 |
| Stuffy ''bad'' air | 242 | 137 | 57 | 31 | 13 | 20 | 8 | 54 | 22 | 84,3 | 67,9 | 78,9 |
| Dust and dirt | 231 | 48 | 21 | 39 | 17 | 18 | 8 | 126 | 55 | 62,7 | 81,6 | 75,3 |
| Noise | 256 | 135 | 53 | 39 | 15 | 32 | 13 | 50 | 20 | 79,2 | 58,5 | 72,3 |
| Restlesness in the classroom | 257 | 147 | 57 | 43 | 17 | 16 | 6 | 51 | 20 | 83,3 | 63,4 | 77,0 |

n(P+S+): number of subjects with positive answer in both self- and parent-administered questionnaires;
n(P-S-): number of subjects with negative answer in both self- and parent-administered questionnaires
n(P-S+): number of subject with negative answer in parent-administered questionnaire and positive answer in self-administered questionnaire
n(P+S-): number of subject with positive answer in parent-administered questionnaire and negative answer in self-administered questionnaire
Ppos: observed proportion of positive agreement; Pneg: observed proportion of negative agreement; Ptotal: observed proportion of total agreement
